# Supplementary material for: Putative Novel Serotypes ‘33’ and ‘35’ in Clinically Healthy Small Ruminants in Mongolia Expand the Group of Atypical BTV
Source: Viruses. 2020 Dec 29;13(1):42. doi: 10.3390/v13010042 (PMC7824028; doi:10.3390/v13010042)
Supplement: Supplementary file 1 [file viruses-13-00042-s001.pdf]

Supplementary materials:

Table S1. Clinical score applied for the experimental inoculation of goats. We modified the score of Saegerman et al., 2008 [1].

Evaluation Group	Symptomatic	Score
**Body temperature**	<40,0°C	0
	≥40°C to <40,5°C	1
	≥40,5°C to <41,0°C	2
	≥41,0°C	3
**General condition**	unremarkable	0
	reduced activity	1
	reduced activity, depressive	2
	Lateral position, clearly reduced activity, apathy	3
**Digestion tract**	unremarkable	0
	Reduced feed or water intake and/or increased salivation	1
	Moderately reduced and/or considerable salivation	2
	Cleary reduced food and water intake and/or strong salivation	3
**Respiration tract**	Unremarkable	0
	Nasal discharge or slight cough	1
	Strong nasal discharge or cough, slight respiratory problems	2
	Strong nasal discharge or cough combined with moderate respiratory problems	3
**Eyes**	Unremarkable	0
	Ocular discharge and/or reddening	1
	Strong ocular discharge and/or strong reddening	2
	Clear conjunctivitis with incrustations	3
**Skin/Mucosa**	Unremarkable	0
	Increased hyperaemia of the hairless skin and mucosa	1
	Single lesions of the mucosa (Nose, mouth)	2
	Increased lesions of the mucosa (Nose, mouth)	3
**Musculoskeletal system**	Unremarkable	0
	Muscle weakness, stiffness	1
	Reddening of the coronary band	2
	Remarkable changes of the coronary band and/or lameness	3
**Swellings and Oedema**	Not present	0
	Slight swellings/Oedema of the head region	2
	Strong swellings/Oedema of the head region	2
	Strong swellings/Oedema of the head region, Protrusion and/or blue coloration of the tongue	3

1. Saegerman, C., A. Mauroy, and H. Guyot, *Bluetongue in ruminants: a standardized clinical report form for the use in different species*, in *Bluetongue in northern Europe*, C. Saegerman, F. Reviriego-Gordejo, and P.P. Pastoret, Editors. 2008, OIE: Paris.

Table S2. Primers and probes of the serotype-specific RT-qPCRs targeting segment 2 for the three Mongolian strains.

Mongolian Strain	Assay Name and Product Size (bp)	Oligo Names	Oligo Sequence (5′-3′)
**BTV-MNG1/2018**	MNG1-Mix3 (119)	BTV-MNG1-130-F	ATT AAA AGT ATA CAT GGC GGC GA
		BTV-MNG1-248-R	CCA ATT TGT CAT CCC CAA TCG TT
		BTV-MNG1-175-FAM	FAM ACT CGT GTA TGC GCT ACC GGT TGC A BHQ1
	MNG1-Mix4 (101)	BTV-MNG1-148-F	GGC GAT ATG CGG AGG GC
		BTV-MNG1-248-R	CCA ATT TGT CAT CCC CAA TCG TT
		BTV-MNG1-175-FAM	FAM-ACT CGT GTA TGC GCT ACC GGT TGC A-BHQ1
**BTV-MNG2/2016**	MNG2-Mix3 (106)	BTV-MNG2-38-F	GCG TAT GGG TCA ATT TGC TAT CA
		BTV-MNG2-143-R	AAT GCA CTT TGA GCG TAT CTA GC
		BTV-MNG2-83-FAM	FAM-TCA CAG GGC GAG GTG AAT TCT TCG A-BHQ1
	MNG2-Mix4 (90)	BTV-MNG2-54-F	GCT ATC ATT ATT ACA CAA ATA CAA TG
		BTV-MNG2-143-R	AAT GCA CTT TGA GCG TAT CTA GC
		BTV-MNG2-83-FAM	FAM-TCA CAG GGC GAG GTG AAT TCT TCG A-BHQ1
**BTV-MNG3/2016**	MNG3-Mix3 (103)	BTV-MNG3-280-F	TAG GTA TCG CTA TTC ACG TAG G
		BTV-MNG3-382-R	CTT ATG CTT CGC TAT ACC GGA
		BTV-MNG3-325-FAM	FAM-CTG TAT AGA TCG AGA TGG CCG ACC C-BHQ1
	MNG3-Mix4 (87)	BTV-MNG3-296-F	CGT AGG CAT ATA CGT GGC GT
		BTV-MNG3-382-R	CTT ATG CTT CGC TAT ACC GGA
		BTV-MNG3-325-FAM	FAM CTG TAT AGA TCG AGA TGG CCG ACC C-BHQ1
